# Are restricted and repetitive behaviours in two‐ and six‐year‐olds associated with emotional and behavioural difficulties?

**DOI:** 10.1002/jcv2.12209

**Published:** 2023-11-03

**Authors:** Sarah J. Carrington, Mirko Uljarević, Elizabeth Meins, Charles Fernyhough, Helen McConachie, Ann Le Couteur, Susan R. Leekam

**Affiliations:** ^1^ School of Psychology College of Health and Life Sciences Aston Universtity Birmingham UK; ^2^ Department of Psychiatry and Behavioral Sciences Stanford University Stanford California USA; ^3^ Department of Psychology University of York York UK; ^4^ Department of Psychology Durham University Durham UK; ^5^ Population Health Sciences Institute Newcastle University Newcastle Upon Tyne UK; ^6^ School of Psychology College of Biomedical and Life Sciences Cardiff University Cardiff UK

**Keywords:** externalising, internalising, restricted and repetitive behaviour

## Abstract

**Background:**

Restricted and repetitive patterns of behaviour (RRBs) serve an adaptive role in development. Elevated levels of RRBs beyond the early years, however, are associated with poorer outcome in language, cognition, and wellbeing, and are seen across a range of neurodevelopmental conditions. This study aimed to characterize the association of distinct RRB subtypes at two and six years of age, with internalising and externalising difficulties in a community sample of children.

**Methods:**

485 parents reported on their child's insistence on sameness (IS) and repetitive sensory and motor (RSM) RRBs at two and six years of age using the Repetitive Behaviour Questionnaire (RBQ‐2). Emotional and behavioural difficulties were measured using the Strengths and Difficulties Questionnaire (SDQ) at age six.

**Results:**

Consistent with previous research, RRBs later in development better predicted emotional and behavioural difficulties at age six than RRBs earlier in development. Moreover, IS RRBs were selectively associated with internalising behaviours and RSM RRBs with externalising behaviours. Importantly, these selective associations depended on when RRBs were measured. Only IS RRBs at age six were significantly associated with internalising behaviour. By contrast, while more RSM RRBs at age six were associated with higher rates of externalising behaviours, higher rates of RSM RRBs at age two were associated with fewer externalising behaviours, adding further support to the previously reported adaptive role of RRBs in early behaviour regulation.

**Conclusion:**

Although there is a need for further research to provide a detailed profile of the adaptive periods for IS and RSM RRBs, the present findings support the potential utility of elevated RRBs as a signal for emotional and behavioural difficulties at age six.

**Key points**

- Although adaptive in early development, elevated levels of restricted and repetitive patterns of behaviour (RRBs) beyond early childhood are associated with poorer outcomes in language, cognition, and wellbeing
- The present study built on previous research to examine the association between distinct RRB subtypes and emotional and behavioural difficulties in a non‐clinical, community sample
- The results provide clear evidence that elevated levels of RRBs beyond adaptive developmental periods are associated with – and may predict – emotional and behavioural difficulties
- Moreover, the findings support the distinctiveness of RRB subtypes, which showed divergent associations with distinct emotional and behavioural difficulties.
- By demonstrating the potential utility of elevated RRBs as a signal for emotional and behavioural difficulties, these findings highlight a potential mechanism for enabling the provision of timely support to reduce the impact of later difficulties.

## INTRODUCTION

Restricted and repetitive behaviours (RRBs) are a multi‐faceted and complex set of behaviours that are a pervasive part of both child and adult experience, spanning behaviours such as rocking and bouncing to repetitive routines, rituals and ‘just right’ behaviours (Evans et al., 1999). RRBs have long been considered an integral part of typical development (e.g. Evans et al., 1997; Leekam et al., 2007), thought to have significance for neural development and voluntary motor control in children from the first year (Leekam et al., 2011; Thelen, 1981), and for mastery, self‐control, and emotional development in the second year (Evans et al., 1999; Kagan, 1981).

Thelen (1979, 1981) originally proposed that while involuntary repetitive motor activities serve an adaptive purpose early in development, when they persist at elevated levels beyond adaptive periods, they may become associated with poorer developmental outcomes and specific difficulties (Thelen, 1981). The presence of elevated levels of RRBs beyond adapitive periods, when they would typically be expected to decline, is associated with neurodevelopmental conditions, including autism spectrum disorders, attention deficit and hyperactivity disorder, and obsessive compulsive disorders (e.g. American Psychiatric Association, 2013; Evans et al., 2017; Leekam et al., 2011), and may reflect a delay in the development of more sophisticated, cognitive mechanisms for emotional and behavioural regulation. Building on studies examining RRBs as risk factors for neurodevelopmental disorder (e.g. Sifre et al., 2021; Wolff et al., 2014), the current study aims to characterize the association of RRBs both during and after adaptive periods, with emotional and behavioural developmental outcomes in a community sample of children.

By definition, community samples likely include individuals with both typical and atypical developmental profiles. Recruiting a community sample, therefore, avoids the circularity inherent in recruiting for, or excluding, specific behaviours, thus enabling associations between RRBs and emotional and behavioural difficulties to be explored in a more neurodevelopmentally representative population. It further allows studies to empirically test the notion that similar mechanisms operate across the full continuum of the trait/symptom distribution (e.g. Cuthbert & Insel, 2013; Kotov, Krueger, Watson, Achenbach, Athoff, Bagby, et al., 2017). If elevated RRBs in specific periods of development are indeed indicative of poorer cognitive, emotional, or behavioural outcomes, this could highlight the timepoint when assessment and support for children is optimal, thus helping to reduce the potential impact of difficulties later. Moreover, transdiagnostic investigations with neurodevelopmentally representative samples have been identified as crucial for developing a better understanding of neurodevelopmental disorder (Astle et al., 2022).

Factor analyses of parent questionnaire data from community samples consistently identify two RRB factors: repetitive sensory and motor (RSM) behaviours, which include hand flapping, rocking, spinning, and fiddling with objects, and insistence on sameness (IS) behaviours, which include routines, rituals, and dislike of changes to particular objects or the environment (e.g. Evans et al., 1997; Leekam et al., 2007). Moreover, these distinct subtypes emerge and decline at different ages (e.g. Sifre et al., 2021). More specifically, RSM behaviours typically emerge in the first year and gradually decline toward the end of the second year of life (Arnott et al., 2010; Evans et al., 1997). In contrast, IS behaviours appear and rapidly increase between the ages of two and four years, after which they decline (Cevikaslan et al., 2014; Uljarević et al., 2017). These two subtypes remain independent across childhood and do not co‐develop; elevated RSM behaviours by age two years predict only RSM and not IS behaviours by age six (Uljarević et al., 2017). This independence in development raises the question of whether RSM and IS subtypes might be differentially related to distinct developmental outcomes at different ages and whether the effect of persistently elevated levels of RRBs may vary depending on the specific RRB subtype.

To date, evidence suggests that RSM and IS behaviours may signal atypical outcomes at different ages. For example, a study by Larkin et al. (2017) using the Repetitive Behaviour Questionnaire‐2 (RBQ‐2; Leekam et al., 2007) found that elevated RSM at 26 months predicted poorer language and social cognition outcomes two years later. The same result was not found for IS behaviours. This result suggests the potential for selective early RSM to signal later language and social cognition outcomes. However, this study measured RRBs at only one time point, when RSM would have peaked and be declining and IS would still be emerging; it is therefore not possible to appraise whether elevated IS behaviours at later ages (i.e., beyond the so‐called ‘typical’ peak) would also predict poorer outcomes.

Other studies also involving community samples of children suggest that IS behaviours at later ages are associated with poorer emotional outcomes, particularly internalizing problems. For example, Laing et al. (2009) reported a significant association between IS and anxiety in 7‐ to 10‐year‐old children. Furthermore, Evans et al. (1999) found that the frequency of fears was more closely correlated with the frequency of IS behaviours in older (48–86 months) rather than younger children (13–48 months). Similarly, Zohar and Bruno (1997) found that although the frequency of childhood rituals (IS behaviours) declined with age in a community sample of 8‐ to 14‐year‐olds, these behaviours were still more closely associated with anxiety in older children than they were in younger children. Other studies with young people with ASD (Lidstone et al., 2014; Rodgers et al., 2012) also showed a selective association between anxiety and IS, but not RSM RRBs. Taken together, these studies indicate that the persistence of IS in later childhood is associated with internalizing problems; however, these studies only assessed IS behaviour at one time point, and thus did not directly examine whether it was only IS later in childhood – as opposed to during more developmentally adaptive periods – that was associated with anxiety. Importantly, these studies did not examine different types of emotional and behavioural difficulties beyond anxiety.

One study that partially addressed this point examined the predictive value of early RRBs for later anxiety in ASD (Baribeau et al., 2020). This study demonstrated a significant association between the RRB severity at diagnosis (between the ages of two and five years) and later anxiety, with some degree of specificity of this association for IS and restricted behaviours. The study did not, however, directly compare the broad constructs of IS and RSM RRBs, nor did it examine internalising behaviours more generally, or include any measure of externalising behaviours. More recently, Keating, Van Goozen, Uljarević, Hay, & Leekam et al. (2023) recruited 4‐ to 8‐year‐old children with non‐specific behavioural and emotional difficulties manifested in school. In this cross‐sectional study, they found that both RSM and IS (measured by the RBQ‐2) were significantly associated with both broad internalising and externalising scores on the Strengths and Difficulties Questionnaire (SDQ; Goodman, 1997), as well as with the specific emotion, conduct, hyperactivity, and peer‐relations subscales.

To our knowledge, only two studies have compared the association between distinct RRB subtypes and emotional and behavioural difficulties beyond anxiety in low‐risk, community samples. In their longitudinal community sample of children aged between 1.5 and 9.5 years, Evans et al. (2014) used the Childhood Routines Inventory (CRI; Evans et al., 1997) to measure RRBs and the Childhood Behaviour Checklist (CBCL; Achenbach & Edelbroack, 1991) to measure emotional and behavioural difficulties. Results showed that for younger typically developing children (mean mental age at Time 1 = 43 months), overall Time 1 RRB scores predicted internalising but not externalising behaviours two years later (Time 2), with no significant findings for distinct RRB subtypes. For older typically developing children (mean mental age at Time 1 = 80 months), overall RRB scores did not predict later internalising or externalising behaviours. For children with Down Syndrome, matched for mental age to the typically developing groups, RRBs were more intense and frequent, and unlike the typically developing children, where RRBs declined with age, IS RRBs remained stable across the study period. Total RRB scores significantly predicted internalising behaviours two years later for younger children with Down Syndrome, while for older children, a selective effect was found, with only scores measuring IS RRBs significantly associating with internalising behaviours. The results for children with Down Syndrome are therefore consistent with the hypothesis that the persistence of higher levels of RRBs later in development may signal poorer outcome. However, this study measured RRBs at only one time point, which was a significant limitation.

The second study examined the association between RRBs and internalising and externalising behaviours in a cross‐sectional community sample of pre‐school children aged between 3.5 and 7 years Ghanizadeh and Moeini (2011) used the Repetitive and Restricted Behaviour Scale (RRBS; Bourreau et al., 2009) as a measure of RRB and the SDQ as a measure of emotional and behavioral problems. The RRBS includes four different sub‐scales: (1) modulation insufficiency (e.g. aggression and stereotyped emotional manifestations); (2) sensorimotor behaviours; (3) reaction to change; (4) restricted behaviours. The sensorimotor subscale is the most closely related to RSM RRBs, whilst both the reaction to change and restricted behaviours subscales relate to IS RRBs; however, the subscales have not been directly mapped to the more typical RSM and IS subtypes. Each of the four RRBS scales was significantly associated with at least one SDQ internalising subscale and at least one externalising subscale. Importantly, no simple association was found between distinct RSM and IS subtypes and the internalising and externalising subscales of the SDQ. However, this cross‐sectional study did not examine distinct longitudinal associations or differential predictive power of RRB subtypes. Importantly, the RRBS had not been validated using factor analysis to establish the consistency of independent subgroups across age, and the construct and face validity of individual subscales is somewhat limited.

The literature reviewed above supports the validity of RSM and IS as distinct RRB subtypes across both normative and atypical development and indicates an association between these RRB subtypes and emotional and behavioural difficulties. However, given the dearth of evidence of both cross‐sectional and longitudinal associations between RSM and IS RRBs with internalising and externalising behaviours, more knowledge is needed. Emotional and behavioural difficulties in childhood can have longlasting impacts on later mental health, school outcomes, and levels of life satisfaction (e.g. Adriaanse, van Domburgh, Zwirs, Doreleijers, & Veling, 2015; Caspi et al., 1996; Hoekstra et al., 2013; Kristoffersen et al., 2015); the identification of reliable early indicators of emotional and behavioural difficulties is therefore important. The current study aimed to capitalize on a unique longitudinal community cohort of children to examine whether the persistence of distinct subtypes of RRBs beyond so‐called adaptive periods was selectively associated with, and predicted emotional and behavioural difficulties. Both IS and RSM were measured using the RBQ‐2, and the SDQ was used to capture both broad constructs of internalising and extrernalising difficulities, as well as their more specific subdomains.

RRBs were measured at two years of age (Time 1), when IS but not RSM would be expected to be prevalent within typical development, and at six years of age (Time 2), when both IS and RSM would be expected to be less prevalent in typical development. Outcomes in relation to emotional and behavioural difficulties were measured at six years. First, we hypothesised a general effect of timing; both RSM and IS at six years would be more strongly associated with emotional and behavioural outcomes than RSM and IS at two years. Second, given previous evidence of the independence of RRB subtypes and their association with anxiety, we hypothesised that IS and RSM would be differentially associated with specific aspects of internalising and externalising difficulties, with IS more strongly associated with internalising behaviours. Finally, if IS and RSM are differentially associated with internalising or externalising outcomes, the pattern of this differential association may depend on when RRBs are measured. Given that RSM would typically have peaked and be on the decline by two years, it was hypothesised that the association between RSM at both time points and externalising behaviours would be significant, but that this association may be larger for Time 2 RSM. By contrast, as elevated IS would be typical at two years, it was hypothesised that only IS at six years would be significantly associated with internalising behaviours.

The study also include important control variables. Previous research indicates that RRBs are associated with socio‐economic status (SES) in community samples (Larkin et al., 2017; Leekam et al., 2007); SES was therefore measured and controlled for in all analyses. Moreover, although evidence of sex differences in RRBs is somewhat limited (for review, see Uljarević et al., 2023), a recent study with 4‐ to 8‐year‐old children referred for assessment of emotional or behavioural difficulties reported that boys had significantly higher rates of both IS and RSM RRBs than girls (Keating et al., 2023). Consequently, sex differences in RRBs and emotional and behavioural outcomes were explored, and sex was also controlled for in analyses.

## MATERIALS AND METHODS

### Participants

Parents of 485 children participated as part of their involvement in one of two longitudinal studies investigating children's health and development in the North‐East of England. The Gateshead Millennium Study (GMS) is a prospective population birth cohort (Parkinson et al., 2007), and the Tees Valley Baby Study (TVBS) is an opportunity‐sampled cohort studied prospectively from eight months. These two studies were conducted in geographically close areas with similar SES levels (Larkin et al., 2017; Parkinson et al., 2007), providing opportunities for combining the samples to study aspects of early development. To assess SES, individual household postcodes were matched to Townsend indices (Townsend et al., 1988), obtained from standardised norms for the North of England using the 2001 census.

Parents of 1237 children (GMS = 1029, TVBS = 208) were recruited, and 485 respondents (360 GMS, 125 TVBS) provided data on RRBs at both Time 1 (two years; 24–33 months) and Time 2 (six years; 70–100 months). The sample was approximately evenly split by sex (224 boys, 235 girls, 8 not specified). The range of SES scores in the sample was −6.5 (very affluent) to 9.1 (very deprived), representing a wide Townsend range. As data were drawn from cohort samples, they overlap with samples reported in several studies from this cohort (e.g. Arnott et al., 2010; Larkin et al., 2017; Leekam et al., 2007; Uljarević et al., 2017). Importantly, the current sample is not identical to samples in previous studies.

### Measures

The Repetitive Behaviour Questionnaire‐2 (RBQ‐2; Leekam et al., 2007) is a 20‐item parent‐report questionnaire designed to record the presence of restricted and repetitive behaviours (RRBs) over the last month. The RBQ‐2 has good psychometric properties, as reported in studies with typically developing 15‐ and 26‐month‐olds from the same cohorts as the current study (Arnott et al., 2010; Leekam et al., 2007), and with a sample of autistic children and adolescents aged 2–17 years (Lidstone et al., 2014). The RBQ‐2 was scored according to procedures reported by Leekam et al. (2007), using a 3‐point scale of severity/frequency, with mean RBQ‐2 scores calculated based on this scale.

The Strengths and Difficulties Questionnaire (SDQ; Goodman, 1997) is a 25‐item questionnaire measuring emotional and behavioural difficulties in children. Scoring and analyses were conducted for the broader internalising and externalising scales and their subscales; internalising (emotional and peer problems) and externalising (hyperactivity and conduct problems). Each item is scored on a three‐point scale, where “0” represents “not true”, “1” is “somewhat true”, and “2” is “certainly true”. In the current study, parents were asked to rate their child's behaviour over the last six months. The SDQ data were collected at Time 2 in both samples.

### Analysis plan

Some questionnaire data were missing for 55 participants. A missing value analysis using Little's Missing Completely at Random test was not significant (*p* > 0.05); however, as 1.5% of cases had missing values for one or more item, missing values were replaced using mean substitution (Tabachnick & Fidell, 2014). Missing data analysis was not conducted for sex; therefore, any analyses that included sex as a variable were conducted on a sample of 477 participants for whom sex was reported.

Bootstrapped bivariate correlations were conducted to explore the association between the two RRB subtypes at both Time 1 and 2, and the SDQ scales and subscales measured at Time 2. Hierarchical linear regression analyses were run to investigate predictors of internalizing and externalizing scales of the SDQ. SES and sex were entered at the first step. Mean scores for the RSM and IS sub‐scales of the RBQ‐2 at Time 1 were entered at the second step, followed by Time 2 RSM and IS scores at the final step. All analyses were bootstrapped with 1000 resamples in order to generate more reliable, robust statistics.

## RESULTS

### Descriptive statistics and preliminary analyses

Mean scores for each assessment are provided in Table 1. The mean scores on the SDQ subscales were all within the normal range indicated by Goodman (1997), and mean RRB scores were comparable with results reported from non‐identical samples from the same cohorts (e.g. Arnott et al., 2010; Leekam et al., 2007; Uljarević et al., 2017).

**TABLE 1:** Mean RBQ‐2 scores at Time 1 and 2, and mean SDQ scores at Time 2.

	Time 1 (2 years)	Time 2 (6 years)
Repetitive, restricted behaviours (RBQ‐2): Mean (SD)		
RSM	1.53 (0.41)	1.25 (0.29)
IS	1.50 (0.38)	1.29 (0.31)
Strengths and difficulties (SDQ): Total (SD)		
Externalising		5.09 (3.55)
Internalising		2.81 (2.70)
Hyperactivity		3.44 (2.51)
Emotional symptoms		1.58 (1.67)
Conduct problems		1.65 (1.58)
Peer problems		1.23 (1.62)

The scores reported for boys and girls (Table S1) were generally comparable, with three exceptions. Mann‐Whitney analyses revealed significantly higher rates of RSM RRBs for boys than girls at Time 2 (boys = 1.30; girls = 1.20; *z* = −3.19, *p* = 0.001). Similarly, boys had higher rates of externalising behaviours than girls (boys = 5.83; girls = 4.37; *z* = 4.37, *p* < 0.001), which appeared to be driven by the hyperactivity subscale (boys = 4.04; girls = 2.85; *z* = 4.87, *p* < 0.001).

Significant, positive correlations (*p* = 0.05/24 = 0.002 following Bonferroni correction) were observed between all scales and subscales of the SDQ and both RSM and IS at Time 2 only (Table 2). Results were largely comparable in boys and girls (Table S2), with positive correlations between Time 2 RRBs and emotional and behavioural difficulties; nevertheless, these correlations did not always survive the correction for multiple comparisons. For example, scores on the internalising scale were significantly correlated with Time 2 IS for both boys and girls, and with RSM for girls. For boys, however, the correlation between internalising behaviours and Time 2 RSM was significant only at a non‐corrected value of *p* < 0.05.

**TABLE 2:** Correlations between repetitive behaviours and emotional and behavioural difficulties.

SDQ	Time 1 (2 years)	IS	Time 2 (6 years)	IS
	RSM		RSM	
Externalising	−0.09	0.00	0.36**	0.21**
Internalising	−0.02	0.04	0.20**	0.28**
Hyperactivity	−0.06	0.02	0.36**	0.17**
Emotional symptoms	−0.01	0.05	0.14*	0.28**
Conduct problems	−0.10	−0.02	0.24**	0.19**
Peer problems	−0.02	0.02	0.19**	0.18**

Abbreviations: IS, insistence on sameness; RSM, repetitive sensory and motor behaviours; SDQ, Strengths and Difficulties Questionnaire.

* = *p*< = 0.002; ** = *p*< = 0.001.

### Externalizing behaviours

Step one of the hierarchical regression (Table 3) included SES and sex, and predicted a significant proportion of the variance (4%) in externalising behaviours (*F*_(2,474)_ = 11.08, *p* < 0.001). Only sex significantly predicted variance, with being male associated with having more externalising behaviours (*t* = ‐4.63, *p* =<0.001, *β* = −0.21). The inclusion of the RSM and IS subscale scores at Time 1 did not significantly improve the model. However, Time 1 RSM behaviours did significantly predict externalising behaviours when IS RRBs, SES, and sex were held constant (*t* = ‐2.23, *p* = 0.013, *β* = −0.11). Moreover, when all other variables were held constant, sex remained significant (*t* = ‐4.61, *p* =<0.001, *β* = −0.21). Including the RSM and IS scores at Time 2 significantly improved the model, (*F*change_(2,472)_ = 30.91, *p* < 0.001), with the final model accounting for 15.4% of the variance in externalising behaviours. When all variables were included, only sex (*t* = ‐3.56, *p* =<0.001, *β* = −0.15) and Time 2 RSM behaviours significantly predicted externalising behaviours (*t* = 6.53, *p* < 0.001, *β* = 0.31). Examination of the standardized *b‐*values (*β*) suggests a change in the nature of the relationship between externalizing behaviours and RSM at two and six years. While RSM at two years was negatively associated with externalising behaviours, indicating that more RSM behaviours were associated with fewer externalising behaviours, the opposite pattern was seen for RSM at six years; that is, more RSM behaviours were associated with more externalising behaviours.

**TABLE 3:** Regression models predicting externalising behaviours.

	Adjusted *R* ^2^	Δ*R* ^2^	*B*	SEB	*β*
Step 1	0.041**				
SES			0.05	0.05	0.05
Sex			−1.47	0.30	−0.21**
Step 2	0.05	0.01			
SES			0.05	0.05	0.05
Sex			−1.46	0.30	−0.21**
Time 1 RSM			−0.97	0.40	−0.11*
Time 1 IS			0.54	0.46	0.06
Step 3	0.15**	0.11**			
SES			0.02	0.05	0.02
Sex			−1.08	0.29	−0.15**
Time 1 RSM			−0.56	0.38	−0.06
Time 1 IS			0.36	0.43	0.04
Time 2 RSM			3.71	0.62	0.31**
Time 2 IS			0.69	0.57	0.06

**p ≤ 0.001; *p < 0.05.

The externalising scale of the SDQ is made up of two sub‐scales (Table 4). Further analysis explored the separate effect of each. As for the externalising scale, the first step of the model for hyperactivity was significant, accounting for 5.5% of the variance (*F*_(2,474)_ = 14.76, *p* < 0.001). Moreover, sex, but not SES, significantly predicted hyperactivity (*t* = ‐5.36, *p* =<0.001, *β* = −0.24). By contrast, this first step was not significant in the model predicting conduct problems. The inclusion of Time 1 RSM and IS scores at Time 2 did not significantly improve the model for either hyperactivity or conduct problems. The inclusion at Time 2 of RSM and IS scores significantly improved both models (hyperactivity: *F*change_(2, 470)_ = 24.46, *p* < 0.001; conduct problems: *F*change_(2,470)_ = 14.69, *p* < 0.001), accounting for an additional 10.4% and 5.8% of the variance respectively. Scores on the conduct subscale were predicted by RSM scores at Time 1 (*t* = −2.25, *p* = 0.018, *β* = −0.12) in step 2 of the model, but in step 3, only Time 2 RSM scores were significant (*t* = 3. 66, *p* < 0.001, *β* = 0.18). Moreover, in line with the findings for the broader externalising scale, conduct problems were negatively associated with RSM at two years and positively associated with RSM at six years. In contrast, scores on the hyperactivity subscale were predicted by scores on the RSM scale at Time 2 only (*t* = 6.82, *p* < 0.001, *β* = 0.32). These findings suggest that the changing pattern of association of RSM RRBs with externalising behaviours between Times 1 and 2 was driven by conduct problems. By contrast, the effect of sex reported for externalising behaviours was driven by the hyperactivity scale, as when all other variables were controlled, sex significant predicted variance in hyperactivity only, in both steps 2 (*t* = ‐5.35, *p* = <0.001, *β* = −0.2) and 3 (*t* = ‐4.33, *p* = <0.001, *β* = −0.19).

**TABLE 4:** Regression models predicting scores on the hyperactivity and conduct problems subscales of the externalising domain.

	Adjusted *R* ^2^	Δ*R* ^2^	*B*	SEB	*β*		Adjusted *R* ^2^	Δ *R* ^2^	*B*	SEB	*β*
Hyperactivity						Conduct problems					
Step 1	0.06**					Step 1	0.00				
SES			0.04	0.03	0.05	SES			0.01	0.02	0.03
Sex			−1.20	0.22	−0.24**	Sex			−0.28	0.15	−0.09
Step 2	0.06	0.01				Step 2	0.01	0.01			
SES			0.04	0.03	0.05	SES			0.01	0.02	0.03
Sex			−1.19	0.22	−0.24**	Sex			−0.27	0.15	−0.08
Time 1 RSM			−0.53	0.28	−0.09	Time 1 RSM			−0.45	0.20	−0.12*
Time 1 IS			0.40	0.35	0.06	Time 1 IS			0.13	0.20	0.03
Step 3	0.16**	0.10**				Step 3	0.07**	0.06**			
SES			0.02	0.03	0.03	SES			0.00	0.02	0.01
Sex			−0.93	0.21	−0.19**	Sex			−0.15	0.14	−0.05
Time 1 RSM			−0.26	0.28	−0.04	Time 1 RSM			−0.30	0.19	−0.08
Time 1 IS			0.30	0.33	0.05	Time 1 IS			0.06	0.19	0.01
Time 2 RSM			2.73	0.42	0.32**	Time 2 RSM			0.98	0.31	0.18**
Time 2 IS			0.16	0.37	0.02	Time 2 IS			0.54	0.29	0.11

**p ≤ 0.001; *p < 0.05.

### Internalising behaviours

The regression model for internalising behaviours is summarised in Table 5. Step one did not account for a significant proportion of the variance. The inclusion of Time 1 RRBs at age two in the second step did not significantly improve the model, accounting for just 0.1% of the variance in internalising behaviour. The inclusion of Time 2 RRBs significantly improved the model (*Fchange*_(2, 470)_ = 21.73, *p* < 0.001), accounting for 8. 1% of the variance. However, only scores on the IS scale at Time 2 significantly predicted internalising behaviours (*t* = 4.88, *p* < 0.001, *β* = 0.24), with more IS RRBs associated with more internalising behaviours.

**TABLE 5:** Regression models predicting internalising behaviours.

	Adjusted *R* ^2^	Δ *R* ^2^	*B*	SEB	*β*
Step 1	0.00				
SES			0.06	0.04	0.08
Sex			−0.04	0.25	−0.01
Step 2	0.00	0.00			
SES			0.06	0.04	0.08
Sex			−0.04	0.25	−0.01
Time 1 RSM			−0.31	0.32	−0.05
Time 1 IS			0.42	0.36	0.06
Step 3	0.08**	0.08**			
SES			0.04	0.04	0.06
Sex			0.12	0.24	0.02
Time 1 RSM			0.01	0.30	0.00
Time 1 IS			0.24	0.35	0.03
Time 2 RSM			0.92	0.60	0.10
Time 2 IS			2.08	0.53	0.24**

**p ≤ 0.001; *p < 0.05.

The two subscales (emotional symptoms and peer problems) that contribute to the internalizing behaviours scale were then examined independently (Table 6). SES and sex did not predict a significant proportion of variance in either subscale. IS and RSM behaviours entered at Time 1 did not significantly explain variance in internalizing behaviours, and were not found to significantly predict scores on either subscale. The inclusion of IS and RSM entered at Time 2 significantly improved the model for both emotional symptoms (*Fchange*_(2,470)_ = 19.89, *p* < 0.001) and peer problems (*Fchange*_(2, 470)_ = 10.84, *p* < 0.001), accounting for 7. 2% and 4. 1% of the variance respectively. As found for the full internalising scale, only scores on the IS and not the RSM subscale at Time 2 predicted scores on the emotional symptom (*t* = 5.39, *p* < 0.001, *β* = 0.26) and peer problem (*t* = 2.52, *p* = 0.048, *β* = 0.13) subscales of the SDQ.

**TABLE 6:** Regression models predicting scores on the emotional and peer problem subscales of the internalising domain.

	Adjusted *R* ^2^	Δ*R* ^2^	*B*	SEB	β		Adjusted *R* ^2^	Δ*R* ^2^	*B*	SEB	*β*
Emotional						Peer problems					
Step 1	−0.00					Step 1	0.01				
SES			0.02	0.02	0.04	SES			0.04	0.02	0.09
Sex			0.07	0.15	0.02	Sex			−0.11	0.15	−0.03
Step 2	0.00	0.01				Step 2	0.00	0.00			
SES			0.02	0.02	0.04	SES			0.04	0.02	0.09
Sex			0.07	0.15	0.02	Sex			−0.11	0.15	−0.03
Time 1 RSM			−0.21	0.20	−0.05	Time 1 RSM			−0.10	0.20	−0.02
Time 1 IS			0.33	0.23	0.07	Time 1 IS			0.10	0.21	0.02
Step 3	0.07**	0.08**				Step 3	0.04**	0.04**			
SES			0.01	0.02	0.02	SES			0.03	0.02	0.08
Sex			0.14	0.15	0.04	Sex			−0.02	0.15	−0.01
Time 1 RSM			−0.03	0.19	−0.01	Time 1 RSM			0.04	0.21	0.01
Time 1 IS			0.22	0.23	0.05	Time 1 IS			0.02	0.20	0.01
Time 2 RSM			0.22	0.31	0.04	Time 2 RSM			0.70	0.36	0.13
Time 2 IS			1.43	0.30	0.26**	Time 2 IS			0.65	0.33	0.13*

**p ≤ 0.001; *p < 0.05.

## DISCUSSION

The present study provides clear evidence of divergent associations between distinct RRB subtypes, assessed at two and six years of age, and emotional and behavioural difficulties at the age of six years in a community sample of children. Although further replication is needed, this research is important as it indicates the potential for a common developmental vulnerability at six years to be indicated by persistently elevated levels of RRBs, possibly enabling the provision of early support.

This study addressed two main questions. The first was whether earlier or later RRBs best predicted emotional and behavioural difficulties. Correlation analyses revealed significant correlations between emotional and behavioural difficulties and RRBs only at Time 2. Similarly, findings from regression analyses indicated that RRBs later in development were the stronger predictors of emotional and behavioural difficulties. Only the inclusion of RRBs at six years of age (Time 2) accounted for a significant proportion of the variance in externalising or internalising behaviours.

Second, it was hypothesised that RSM and IS subtypes would be differentially related to behavioural and emotional difficulties. Correlation analyses revealed significant positive correlations between both IS and RSM RRBs at Time 2 and each of the internalising and externalising scales and their constituent subscales. These results are consistent with findings reported by Keating et al. (2023), who argued this correlation supported a potential role for RRBs as a broader measure of mental health and wellbeing in a non‐clinical sample. However, results from the regression analyses in this community sample showed that when controlling for all other variables – including other RRBs – only IS and not RSM significantly predicted variance in the broad internalising construct and its specific emotional problems and peer problems subscales. In contrast, RSM showed a selective association with the broad externalising construct, as well as with the hyperactivity and conduct problems subscales.

Consistent with the finding that boys had significantly higher scores than girls on the externalising and hyperactivity scales, sex also significantly predicted variance in these behaviours. Sex remained significant in each step of the model, although in the final step, Time 2 RSM RRBs were the strongest predictor. Time 2 RSM RRBs were also significantly more common in boys than girls in this sample. There is limited evidence of sex differences in RRBs, with a recent review suggesting that those studies that do report sex differences in RRBs in autism typically report higher rates in males (Uljarević et al., 2023). Nevertheless, the current result of sex differences in RSM but not IS RRBs is inconsistent with findings from a non‐clinical sample of children with emotional and behavioural difficulties, in which higher rates of both IS and RSM RRBs were reported for males (Keating et al., 2023).

The potential reasons for the distinct pattern of associations between RRB subtypes and emotional and behavioural difficulties revealed in the regression analyses are intriguing. Although an association between IS and anxiety or fears has been reported in both clinical and non‐clinical samples (e.g. Evans et al., 1999; Laing et al., 2009; Lidstone et al., 2014; Zohar & Bruno, 1997), the nature of that association is still to be clarified. As previously discussed, it has been suggested that the persistence of IS beyond adaptive periods may serve to reinforce anxiety (i.e. anxiety as a consequence of IS). For example, South and Rodgers (2017) presented a model of ASD in which they suggest that uncertainty over whether rules or routines would be adhered to may lead to greater anxiety. An alternative explanation, however, may be that heightened levels of anxiety reinforce reliance on IS behaviours, such as insistence on adherence to routines and sameness in the environment, in an effort to reduce unpredictability and uncertainty; that is, IS as a consequence of anxiety. Consistent with the latter argument, Hwang et al. (2019) reported that intolerance of uncertainty mediated the association between IS and anxiety in a sample of autistic adults.

Time 2 RSM were also positively correlated with internalising behaviours, and may reflect consequences of anxiety, such as engaging in repetitive motor mannerisms (or stimming) to self‐soothe. Nevertheless, these behaviours did not significantly predict internalising behaviours when IS were included in the model. Instead, RSM significantly predicted externalising behaviours, even when IS were controlled. The hyperactivity scale appeared to drive the effects seen for the broader externalising scale, and given the association between features of hyperactivity and impulsivity described in the DSM‐5 criteria for ADHD (American Psychiatric Association, 2013), this is perhaps not surprising.

A subsidiary question linked to the differential relation between RRB sybtypes and emotional and behavioural difficulties described above was whether the nature of the relation would be dependent on the age at which RRBs were measured. RSM behaviours at both two (Time 1) and six (Time 2) years of age were significantly associated with externalising behaviours, both with the broader externalising domain and the conduct problems subscale, although Time 1 RRBs were no longer significant when Time 2 RRBs were included in the model. This result initially appears to support previous findings. In typical development, RSM behaviours reach their peak between 12 and 15 months (Arnott et al., 2010) and as such, elevated rates of RSM at two years may be indicative of later problems, while lower rates may suggest more ‘adaptive’ development. More detailed examination of the regression analyses in the present study for externalising behaviours and conduct problems, however, pointed to an apparent paradox. Specifically, while RSM at six years were positively associated with externalising and conduct problems at six years, suggesting – as predicted – that higher rates of RSM beyond the adaptive period at age six were associated with poorer outcome, earlier RSM were significantly negatively associated with later externalising and conduct problems; that is, higher rates of RSM RRBs at the age of two were associated with fewer externalising and conduct problems. This latter finding is intriguing. One possible explanation might relate to the proposed adaptive role of early RSM in regulating behaviour (Thelen, 1981), which may last longer than anticipated. Further research measuring RRBs at more time points across development, along with measures of language and cognition, would be required to explore the proposed adaptive period for RSM further.

Unlike RSM RRBs, only Time 2 (age six) IS were significantly associated with internalising behaviours. The so‐called adaptive period for IS extends for longer, with levels reported to peak around four years of age (Cevikaslan et al., 2014; Evans et al., 1997), and previous studies have reported that while early IS might serve an adaptive purpose to alleviate fears and anxiety, the persistence of these behaviours may later come to reinforce fears and anxiety (Evans et al., 1999; Keating et al., 2023; Zohar & Bruno, 1997). In the present study, the Time 2 measurement was within the period in which IS RRBs would typically be expected to decline. Further information about IS RRBs levels in general population samples as children approach school entry (in the UK, from age four years upwards) would enable investigation of whether it is the continuation of RRBs beyond the so‐called adaptive period that is associated with later emotional and behavioural difficulties.

### Strengths and limitations

The findings reported here should be considered in the light of several limitations. First, although RRBs were measured at two time points, this is still a relatively sparse representation of development, which limits conclusions that can be drawn about the developmental trajectory. Moreover, the SDQ was measured at only one time point. In future, the developmental shift in the relationship between RRBs and emotional and behavioural difficulties could be further investigated using a higher frequency ‘sampling’ frame within a longitudinal design to identify the developmental trajectories of RRBs beyond the first few years of life and potentially identify the earliest point from which RRB subtypes predict emotional and behavioural difficulties in mid‐ to late‐childhood.

Second, both RRBs and emotional and behavioural difficulties were measured using parent‐report questionnaires. Questionnaires are reliable and valid measures of RRB, capturing behaviours across different contexts, including behaviours that may be less likely to be captured in observational protocols. Nevertheless, using information from multiple sources or informants to provide a more detailed view of a child's behaviour in multiple contexts, could mitigate potential shared method variance. The inclusion of teacher‐report questionnaires, and in particular observational measures of both RRBs and the emotional and behavioural problems captured by the SDQ, could further strengthen research in this field.

Third, it could be argued that the apparently weak association between early RRBs and later behaviour reflected measurement error or bias. While Time 1 RRBs were measured at the age of two years, Time 2 RRBs were measured at the same time as the SDQ, when children were six years of age. It is possible, therefore, that the stronger association between the SDQ and RRBs at six years simply reflects the fact that parents were reporting on these behaviours at the same time. However, the findings of significant associations between both Time 1 and Time 2 RSM and both conduct problems and externalising behaviours in general does not support this interpretation. Nevertheless, associations were not found for early IS and internalising behaviours.

Finally, it should be noted that although RRBs accounted for a significant proportion of the variance in emotional and behavioural difficulties, the proportion accounted for in any of the regression models was relatively small. For example, although significant, the inclusion of SES, sex, and RRBs at both time points accounted for just 15% of the variance in externalising behaviours. As such, RRBs are clearly not the only significant factor in the development of emotional and behavioural difficulties.

## CONCLUSION

This study represents an important step forward in our understanding of the association between distinct RRB subtypes and emotional and behavioural outcomes. The large sample was drawn from two community samples with a broad SES range suggesting a good representation of the general population. Moreover, both the SDQ and the RBQ‐2 scores for the sample were within the expected range for a general population sample (Goodman, 1997; Leekam et al., 2007; Uljarević et al., 2017) and, importantly, lower than mean scores reported for children referred for neurodevelopmental assessment (Keating et al., 2023). As such, the findings reported here are likely representative of the general population.

The measurement of RRBs at two time points and the use of regression analysis enabled examination not only of the association of early and later RRBs with emotional and behavioural outcomes but also facilitated investigation of the predictive power of RRBs for these outcomes. This, combined with the use of established subtypes of RRBs and analysis of the broad internalising and externalising constructs thought to be most appropriate for low‐risk samples (i.e. general population; Goodman et al., 2010; Goodman & Goodman, 2009), directly addressed limitations identified in the two previous studies examining associations between RRBs and internalising and externalising behaviours.

This study provides clear evidence that elevated levels of RRBs beyond developmental periods that are typically considered adaptive are associated with – and may predict – emotional and behavioural difficulties. Moreover, the findings in this study lend further support to the distinctiveness of RRB subtypes, with divergent associations between IS and RSM RRBs and internalising and externalising behaviours respectively. Although there is a need for further research to provide a detailed profile of the adaptive periods for IS and RSM RRBs, the present findings support the potential utility of elevated RRBs as a signal for emotional and behavioural difficulties.

## AUTHOR CONTRIBUTIONS

**Sarah J. Carrington**: Conceptualization; formal analysis; methodology; writing – original draft; writing – review & editing. **Mirko Uljarević**: Conceptualization; methodology; writing – review & editing. **Elizabeth Meins**: Conceptualization; data curation; funding acquisition; methodology; writing – review & editing. **Charles Fernyhough**: Conceptualization; data curation; methodology; writing – review & editing. **Helen McConachie**: Conceptualization; data curation; methodology; writing – review & editing. **Ann Le Couteur**: Conceptualization; methodology; writing – review & editing. **Susan R. Leekam**: Conceptualization; data curation; funding acquisition; investigation; methodology; writing – review & editing.

## CONFLICT OF INTEREST STATEMENT

The authors have declared that they have no competing or potential conflicts of interest.

## ETHICS STATEMENT

Ethical approval was obtained from Gateshead, South Tees, Hartlepool and North Tees, County Durham and Darlington Local Primary Health Care Research Ethics Committees. All respondents provided informed consent.

## Supporting information

Supporting Information S1

## Data Availability

Data sharing is not applicable to this article as no new data were created or analysed in this study.
