# Supplementary material for: Are restricted and repetitive behaviours in two‐ and six‐year‐olds associated with emotional and behavioural difficulties?
Source: JCPP Adv. 2023 Nov 3;4(3):e12209. doi: 10.1002/jcv2.12209 (PMC11472799; doi:10.1002/jcv2.12209)
Supplement: Supplementary file 1 — Supporting Information S1 [file JCV2-4-e12209-s001.docx]

**Supporting Information**

| Table S1  *Mean RBQ-2 scores at Time 1 and 2, and mean SDQ scores at Time 2 for males (n=242) and females (n=235)* | | | | | | |
| --- | --- | --- | --- | --- | --- | --- |
|  | **Time 1 (2 years)** | | | **Time 2 (6 years)** | | |
|  | **Boys** | **Girls** |  | **Boys** | **Girls** |  |
| ***Repetitive, restricted behaviours (RBQ-2):* Mean (SD)** |  |  |  |  |  |  |
| RSM | 1.51 (.40) | 1.55 (.41) | *U*=27443, *Z*=-.66 | 1.30 (.33) | 1.20 (.25) | *U*=23744.50, *Z*=-3.91** |
| IS | 1.48 (.37) | 1.51 (.38) | *U*=27142, *Z*=-.86 | 1.31 (.35) | 1.27(.26) | *U*=28091.50, *Z*=-.23 |
|  |  |  |  |  |  |  |
| ***Strengths and difficulties (SDQ):* Total (SD)** |  |  |  |  |  |  |
|  |  |  |  | **Boys** | **Girls** |  |
| Externalising |  |  |  | 5.83 (3.71) | 4.37 (3.19) | *U*=21883, *Z*=-4.37** |
| Internalising |  |  |  | 2.82 (2.91) | 2.80 (2.51) | *U*=27427, *Z*=-.68 |
|  |  |  |  |  |  |  |
| Hyperactivity |  |  |  | 4.04 (2.65) | 2.85 (2.19) | *U*=21162, *Z*=-4.87** |
| Emotional symptoms |  |  |  | 1.54 (1.75) | 1.62 (1.61) | *U*=26829, *Z*=-1.10 |
| Conduct problems |  |  |  | 1.79 (1.66) | 1.52 (1.49) | *U*=25908, *Z*=-1.72 |
| Peer problems |  |  |  | 1.28 (1.77) | 1.18 (1.46) | *U*=28299, *Z*=-.10 |

*=p<.05; **=p<.001

| **Table S2** |  | | | |  | | | |
| --- | --- | --- | --- | --- | --- | --- | --- | --- |
| *Correlations between repetitive behaviours and emotional and behavioural difficulties for males (n=242) and females (n=235)* | | | | | | | | |
|  | **Time 1 (2 years)** | | | | **Time 2 (6 years)** | | | |
|  | **RSM** | | **IS** | | **RSM** | | **IS** | |
| **SDQ** | **Boys** | **Girls** | **Boys** | **Girls** | **Boys** | **Girls** | **Boys** | **Girls** |
| Externalising | -.05 | -.13 | .01 | .01 | .41** | .25** | .22** | .18 |
| Internalising | .00 | -.04 | -.01 | .10 | .18 | .24** | .29** | .27** |
|  |  |  |  |  |  |  |  |  |
| Hyperactivity | -.02 | -.10 | .01 | .05 | .41** | .23** | .17 | .15 |
| Emotional symptoms | .00 | -.04 | .00 | .10 | .12 | .21* | .30** | .26** |
| Conduct problems | -.08 | -.12 | .00 | -.04 | .26** | .20* | .21** | .16 |
| Peer problems | .00 | -.02 | -.02 | .06 | .18 | .18 | .18 | .18 |

*p<=.002; **p<=.001; SDQ=Strengths and Difficulties Questionnaire; RSM=repetitive sensory and motor behaviours; IS= insistence on sameness
